# An effective photothermally active titanium-copper nanocomposite for breast cancer therapy

**DOI:** 10.1039/d6ra04493j

**Published:** 2026-07-07

**Authors:** Laleh Salarilak, Abolfazl Doosti, Mehdi Haddad, Ali Kalantari-Hesari, Faezeh Almasi, Farnaz Eslami, Kiana Nazari Nasi, Mir-Jamal Hosseini, Aziz Maleki

**Affiliations:** a Zanjan Pharmaceutical Nanotechnology Research Center (ZPNRC), Zanjan University of Medical Sciences Zanjan 45139-56184 Iran; b Department of Pharmaceutical Nanotechnology, School of Pharmacy, Zanjan University of Medical Sciences Zanjan 45139-56184 Iran maleki@zums.ac.ir; c Student Research Committee, School of Pharmacy, Zanjan University of Medical Sciences Zanjan 45139-56184 Iran; d Department of Basic Sciences, Faculty of Veterinary Medicine, Bu-Ali Sina University Hamedan Iran; e Zanjan Applied Pharmacology Research Center, Health and Metabolic Diseases Research Institute, Zanjan University of Medical Sciences Zanjan Iran jamal_hossini@yahoo.com

## Abstract

Cancer therapy faces several challenges including metastasis, subsequent recurrence and the high susceptibility of immunocompromised patients to secondary bacterial infections. In this study, a copper-doped titanium dioxide nanocomposite (Ti-Cu NC) was developed as a multifunctional photothermal agent for breast cancer treatment. In 4T1 murine breast cancer cells, Ti-Cu NCs induced significant (approximately 3.8-fold increase compared to control) reactive oxygen species (ROS) production, leading to apoptosis and cell death with a synergistic effect observed upon near-infrared (NIR) irradiation (1.5 W cm^−2^, 10 min). The nanocomposites also reduced the migratory capacity of 4T1 cells, indicating potential anti-metastatic activity. Importantly, Ti-Cu plus NIR irradiation triggers immunogenic cell death (ICD) markers in cancer cells, suggesting the ability to activate host antitumor immunity against residual and metastatic cancer cells. Beyond anticancer effects, the synergistic photothermal activity of Ti-Cu NCs effectively suppressed bacterial growth. Antibacterial rates of 99.57%, 99.4%, 99.6%, and 98.3% were recorded for MRSA, *K. pneumoniae*, *E. coli*, and *P. aeruginosa*, respectively, at a concentration of 300 µg mL^−1^. At a concentration of 1000 µg mL^−1^, the antibacterial rate was 100% for all four pathogenic strains in the presence of NIR, addressing the clinical challenge of cancer-related infections. *In vivo*, Ti-Cu NCs combined with NIR irradiation demonstrated potent anticancer efficacy (temperature rise to ∼44.5 °C, significant tumor growth inhibition) with no detectable toxicity following intraperitoneal injection, even at higher doses. These findings establish Ti-Cu NCs as a safe, biocompatible, and dual-functional platform that induces ROS-mediated apoptosis, suppresses tumor cell migration, triggers immunogenic cell death, and eliminates bacteria *via* photothermal synergy, highlighting their potential as promising candidates for integrated cancer immunotherapy and infection control.

## Abstract 2

Ti-Cu NCs are constructed and upon NIR irradiation, generate a potent photothermal effect, which induces immunogenic cell death (ICD) in 4T1 tumor cells and are an effective cancer treatment in a mouse model.

## Graphical abstract

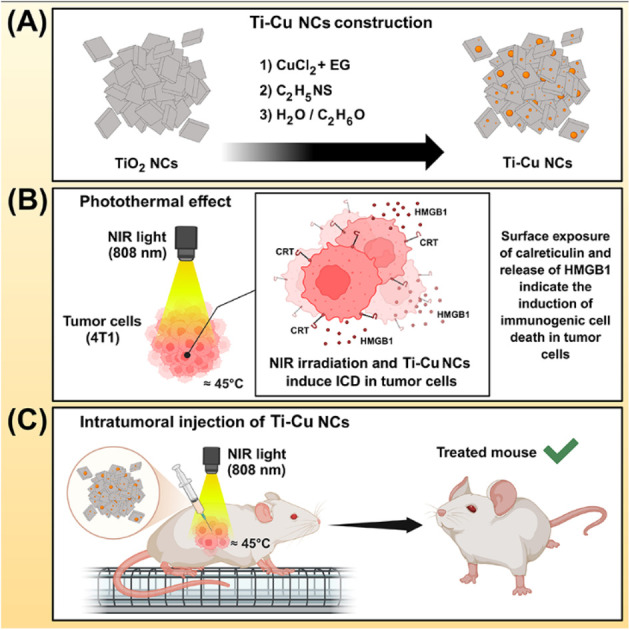

## 1. Introduction

Cancer remains one of the most significant threats to global health.^1^ Common treatment methods, like surgery, chemotherapy, and radiotherapy have limitations such as invasive procedures, systemic toxicity, damage to healthy tissues, and limited efficacy against metastatic or recurrent disease.^2,3^ In addition, current therapies fail to simultaneously address tumor ablation, metastatic spread, immunosuppression, and secondary bacterial infections in immunocompromised patients. Photothermal therapy (PTT) has emerged as a promising alternative.^4^ In this method, photothermal agents (PTAs) convert near-infrared (NIR) light energy into localized heat. Induced hyperthermia eliminates cancerous cells with spatio-temporal selectivity while protecting surrounding healthy tissues.^5^ However, a single photothermal platform that integrates photothermal therapy, immune system activation, and broad-spectrum antibacterial activity is still lacking.

**Scheme 1:**
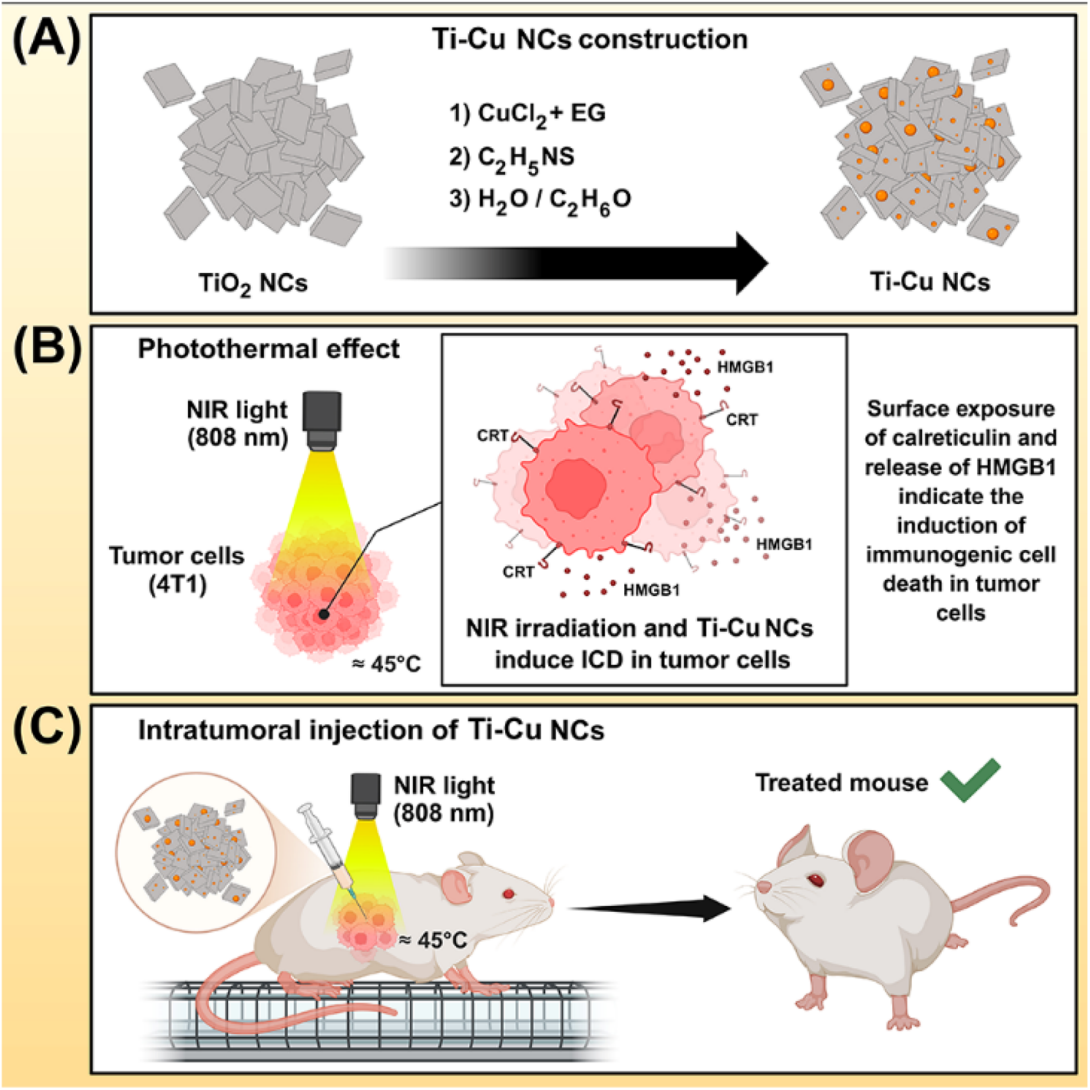
Schematic representation of the fabrication and therapeutic application of Ti-Cu. (A) Synthesis of Ti-Cu NCs. (B and C) Photothermal effect of Ti-Cu NCs followed by NIR irradiation induces ICD and tumor ablation in breast cancer model.

PTT performance is critically dependent on the performance of the PTAs. An ideal PTA must combine high photothermal conversion efficiency, biocompatibility and photostability.^6,7^

Titanium dioxide (TiO_2_) is widely used in pharmaceutical industries due to its established biocompatibility and chemical stability.^8,9^ As a potent photocatalyst and through photo-induced electron transfer processes,^10^ stimulated TiO_2_ can react with water and oxygen to produce reactive oxygen species (ROS) and induce cancer cell death.^11,12^ However, its inherent wide bandgap (∼3.2 eV) limits light absorption to the ultraviolet spectrum, limiting its biomedical application. Several methods, such as metal doping, have been proposed to improve photocatalytic activity of TiO_2_ under visible light. Integration of copper into the TiO_2_ nanosheets addresses this fundamental limitation through bandgap engineering.^13^ Specifically, copper doping introduces intermediate energy states, narrowing the bandgap and extending light absorption into the visible and NIR regions and enhances the photocatalytic activity of TiO_2_.^14^

In addition, due to tumor-induced immunosuppression, disruption of mucosal barriers by chemotherapy or radiotherapy and medical devices cancer patients are at risk of infectious complications.^15^ Bacterial colonization and biofilm formation cause sepsis and chronic inflammation that can paradoxically promote tumor progression. Importantly, multidrug-resistant organisms such as methicillin-resistant *Staphylococcus aureus* (MRSA), *Pseudomonas aeruginosa*, *Escherichia coli*, and *Klebsiella* species mainly *Klebsiella pneumoniae* are frequently isolated from cancer patients with bloodstream infections.^15^ Therefore, there is an urgent need for therapeutic platforms that simultaneously address both malignancy and associated microbial infections, particularly in immunocompromised hosts.

Previous studies have reported copper-doped TiO_2_ nanocomposites for various applications. For example, copper-doped TiO_2_ nanocomposites coated with Pluronic® F-127 were used against cancer cells. Upon UV irradiation, nanocomposites generated ROS, leading to 90% toxicity against 4T1 breast cancer cells, demonstrating selective photodynamic therapy. The same nanocomposites also exhibited significant antibacterial activity against *Escherichia coli* and *Staphylococcus aureus* compared to pure TiO_2_.^16^ However, these systems relied on UV activation (clinically impractical due to poor tissue penetration) and did not evaluate ICD or anti-metastatic effects.

Unlike previously reported Ti-Cu systems, which rely primarily on UV or visible light activation^16,17^ and thus suffer from poor tissue penetration and limited biomedical applicability, the present Ti-Cu NC is activated by near-infrared (NIR) light at 808 nm, enabling deeper tissue penetration^18^ and more effective photothermal therapy. Moreover, previous Cu-TiO_2_ nanocomposites have not systematically evaluated key therapeutic parameters such as suppression of cancer cell migration, induction of immunogenic cell death (ICD) in terms of activation of host's immune system against residual and metastatic cancer cells.

This work evaluates NIR-triggered, ROS-mediated apoptosis, immunogenic cell death (ICD) induction, and broad-spectrum antibacterial activity within a single Ti-Cu nanocomposite platform specifically for breast cancer therapy and post-treatment infection control in a single platform (Scheme 1).

## 2. Materials and methods

### 2.1. Materials

Tetrabutyl orthotitanate, hydrofluoric acid, ethanoland formalin were purchased from Merck company. CuCl_2_·2H_2_O and ethylene glycol were purchased from Sigma-Aldrich company. The chemicals were of analytical grade and used without further purification.

### 2.2. Preparation of Ti-Cu NCs

Titanium dioxide (TiO_2_) nanocomposites were synthesized using a hydrothermal method.^19^ 10 mL of tetrabutyl orthotitanate (TBOT, 98%) was mixed with 1.4 mL of 47% hydrofluoric acid (HF) in a 50 mL Teflon-lined stainless steel autoclave. The sealed autoclave was heated at 180 °C for 24 hours and then allowed to cool naturally to room temperature. The resulting white precipitate was collected by centrifugation at 3500 rpm and washed three times with deionized water followed by three times with absolute ethanol. The washed TiO_2_ nanosheets were dried at room temprature for 12 hours. For the synthesis of the Ti-Cu nanocomposite, 0.17 g (1 mmol) of CuCl_2_·2H_2_O was dissolved in 40 mL of ethylene glycol and sonicated for 15 minutes. Then, 50 mg of the pre-synthesized TiO_2_ nanosheets was added and dispersed by sonication for 30 minutes. Over 3 minutes, 0.113 g (1.5 mmol) of thioacetamide was added slowly under continuous stirring inside a fume hood. After stirring vigorously for 2 hours, the mixture was transferred to a 50 mL Teflon-lined autoclave and heated at 180 °C for another 24 hours. The final dark product (Ti-Cu NC) was collected by centrifugation at 3700 rpm, washed five times with deionized water and three times with absolute ethanol and dried at room temperature for 24 hours before storage.

### 2.3. Characterization of Ti-Cu NCs

The morphology, microstructure, and elemental content of Ti-Cu NCs were analyzed by Transmission Electron Microscopy (TEM) (Philips EM208S, Netherlands) and Scanning Electron Microscopy (Quanta 250 FEG, USA). Atomic force microscopy (AFM) Park Systems XE-100 AFM (Park Systems, Korea) were employed to elucidate the nanoscale structure and surface topography. Energy-dispersive X-ray spectroscopy (EDXS) was utilized to determine the elemental composition and distribution, while attenuated total reflectance Fourier transform infrared spectroscopy (ATR FTIR) Bruker Tensor 27 ATR-FTIR (Bruker, Germany) was used to analyze the chemical bonding and surface functional groups. The UV-vis-NIR analysis of NPs was studied by a UV-vis spectrophotometer (Bruker IFS, 66/vs).

### 2.4. Reactive oxygen species detection

The evaluation of hydroxyl radical (˙OH) production was conducted *via* a methylene blue (MB) degradation assay.^20^ Specifically, a solution containing Ti-Cu NCs (300 µg mL^−1^), H_2_O_2_ (200 µL, 10^−3^ M), and MB (1 mL, 0.001 M) was exposed to 808 nm near-infrared light at intensities of 1 or 1.5 W cm^−2^ for a duration of 30 minutes. The degradation of MB, indicative of °OH formation, was subsequently determined by spectrophotometric measurement of absorbance at 652 nm. Measurements were conducted in triplicate.

### 2.5. Photothermal performance measurement

The photothermal performance of Ti-Cu NCs was evaluated using 500 µL of aqueous dispersion at concentrations of 300 and 600 µg mL^−1^. The samples were placed in a quartz cuvette and irradiated with an 808 nm NIR laser diode (3L-IR, Hamerz Rad) at power densities of 1.0 and 1.5 W cm^−2^ for 10 min. The laser beam was positioned vertically at a fixed distance of 1 cm from the sample surface, producing a circular irradiation spot with a diameter of approximately 0.5 cm. The sample was placed at the center of the laser spot to ensure uniform irradiation. Temperature changes during irradiation were monitored using the near infrared camera, which was positioned at a distance of 1 cm from the sample. The temperature of the irradiated region was recorded during the irradiation period. The photothermal stability of Ti-Cu NCs at 300 µg mL^−1^ was further investigated through five consecutive laser on/off irradiation cycles under the same irradiation conditions. The photothermal conversion efficiency of Ti-Cu NCs was calculated according to previous study.^21^

### 2.6. Cell viability assay

The 4T1 (metastatic adherent epithelial murine breast cancer cells) were purchased from Royan Institute of Iran and were cultured in Dulbecco's Modified Eagle Medium (DMEM) supplemented with 10% fetal bovine serum and 1% penicillin-streptomycin at 37 °C in a 5% CO_2_ atmosphere. The cytotoxic effects of Ti-Cu NCs were evaluated using the 3-(4,5-dimethylthiazol-2-yl)-2, 5-diphenyltetrazolium bromide (MTT) assay.^22^ Briefly, 0.6 ×10^4^ cells per well were placed in each well of a 96-well plate. Upon reaching 90% confluence, the media were extracted and the cells were subjected to different concentrations of Ti-Cu NCs with or without NIR irradiation (1.5 W cm^−2^, 5 min). Subsequently, the media were aspirated and then a diluted solution of MTT (5 mg mL^−1^) was added, followed by another incubation for 4 hours at 37 °C to allow the formation of formazan crystals. Afterward, the produced formazan crystals were solved in 150 µL of dimethyl sulfoxide (DMSO) and the absorbance at 570 nm was measured using a microplate reader (BioTek). The cell viability percentage was determined using the following formula:
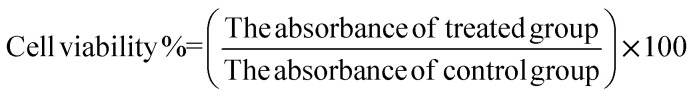


### 2.7. Cell apoptosis assays

Cell apoptosis was evaluated by Annexin V-FITC and propidium iodide co-staining of the 4T1 cells and flow cytometry method.^23^ 0.6 ×10^4^ cells in each well of a 96-well plate were seeded. After reaching the desired confluency, cells were treated with fresh media containing 250 µg mL^−1^ of Ti-Cu NCs, with or without NIR irradiation (1.5 W cm^−2^, 5 min). The control group remained untreated. Each experimental group was replicated three times. After a 3 hour incubation period, the media were removed, and cells from each group were washed with buffered saline (PBS), harvested carefully and then incubated for 15 minutes in 500 µL of binding buffer provided by the kit (Rosh Azma apoptosis detection kit). Then 3 µL of Annexin V-FITC and 1.5 µL of propidium iodide were added at room temperature in the dark. Subsequently, the cells were subjected to flow cytometry analysis using a flow cytometer (BD Accuri C6 plus). FlowJo 10 software was utilized for data analysis.

### 2.8. *In vitro* cell migration assay

For cell migration assay, 0.8 × 10^4^ cells per well 4T1 cells were cultured in 24-well plates and allowed to form a monolayer. A 200 µL sterile tip was used to scratch a vertical wound, cellular debris were removed by washing the cells with PBS twice. Then, the culture media were replaced with fresh media in the control group, and fresh media containing Ti-Cu NCs at a concentration of 250 µg mL^−1^ in other groups with or without NIR irradiation (1.5 W cm^−2^, 5 min). Next, the cells were imaged, and photography was continued every 24 hours until the gap in the control group was filled. The area of gap filling was quantified using ImageJ software.^24^

### 2.9. Intracellular ROS detection

The intracellular ROS detection was performed with reactive oxygen species fluorometric assay Kit (Kiazist, Iran).^25^ In brief, 0.8 × 10^4^ cells were seeded into each well of a black 96-well plate. Confluence cells were incubated with 150 µL DCFDA reagent (20 µM) in the dark for 45 min before treatment. Then, cells were treated with 250 µg mL^−1^ of Ti-Cu NCs with or without NIR irradiation (1.5 W cm^−2^, 5 min). Afterward, the fluorescence intensity of ROS induced oxidized DCFDA in cells was measured in fluorescence microplate reader (BioTek) in *E*_x_/*E*_m_ = 485/535 nm.

### 2.10. Cellular uptake analysis by flow cytometry

Cellular internalization of the Ti-Cu NCs was evaluated *via* flow cytometry by measuring the characteristic increase in cellular side scatter (SSC) upon nanoparticle uptake.^26^ 4T1 cells were seeded in 96-well plates and allowed to adhere overnight. Cells were then treated with fresh culture medium containing well-dispersed Ti-Cu NCs at a concentration of 250, 150 and 50 µg mL^−1^.

Following incubation periods of 1 and 3 hours, the cells were washed twice with PBS to remove any non-internalized nanoparticles, trypsinized, and resuspended in PBS for immediate analysis. Untreated cells were processed in parallel as a control. All samples were analyzed using a BD FACS accuri 6 plus flow cytometer, with the SSC signal collected in the standard configuration. The geometric mean of the SSC signal was recorded for a minimum of 10 000 single-cell events per sample. A definitive rightward shift in the SSC histogram, corresponding to an increase in the granularity/internal complexity of the cells due to nanoparticle uptake, was used to confirm and quantify cellular internalization. Data from three independent experiments were analyzed.

### 2.11. *In vitro* analysis of ICD hallmarks: surface calreticulin exposure and extracellular HMGB1 release

Extracellular release of HMGB1 into the supernatant of treated cells was assessed by ELISA. Culture supernatants of treated cells (Ti-Cu NCs with or without NIR) were collected, centrifuged at 300 g for 5 minutes to remove any cellular debris, and stored at −80 °C until analysis. Extracellular HMGB1 concentration was quantified using a commercial Mouse HMGB-1 ELISA Kit (Elabscience®, Catalog No. E-EL-M0763) according to the manufacturer's instructions. Absorbance was measured at 450 nm using a microplate reader, and HMGB1 concentrations (pg mL^−1^) were calculated from a standard curve generated with the provided recombinant protein.

For the analysis of calreticulin (CRT) surface exposure, cells were harvested immediately post-treatment using trypsinization. The harvested cells were transferred to appropriate tubes and washed with ice-cold PBS containing 2% FBS. This washing step involved centrifugation at 300 g for 5 minutes at 4 °C. The supernatant was carefully discarded, and the cell pellet was resuspended in the same ice–cold PBS/FBS buffer. Following a cell count, the concentration was adjusted to 0.5–1 × 10^6^ cells per staining tube. These prepared cells were then incubated for 30 minutes at 4 °C in the dark with a FITC-conjugated calreticulin polyclonal antibody (Proteintech, Catalog No. 27298-1-AP; dilution 1 : 100) or a matching FITC-conjugated isotype control. After incubation, cells were washed twice with the ice-cold PBS/FBS buffer to remove unbound antibody, fixed in PBS containing 1% paraformaldehyde, and analyzed immediately on a flow cytometer. A minimum of 10 000 single-cell events were acquired per sample, and the percentage of CRT positive cells was determined by gating on the live cell population and comparing the fluorescence intensity to the isotype control.

### 2.12. *In vitro* antibacterial activity

#### 2.12.1 Bacterial culture condition

*Escherichia coli* (*E. coli*, ATCC 25922), *Klebsiella pneumoniae* (*K. pneumoniae*, ATCC 13883), *Pseudomonas aeruginosa* (*P. aeruginosa*, ATCC 25668) and methicillin-resistant *Staphylococcus aureus* (MRSA, ATCC25923) were used to investigate the antibacterial performance of Ti-Cu nanoparticles. Briefly, the bacteria were cultured in Mueller–Hinton broth (MHB) at 37 °C and 120 rpm overnight until reached the optical density at 600 nm of 1. The bacterial suspension was diluted with MHB medium to the concentration equal to 5 × 10^5^ CFU mL^−1^ for antibacterial assays.

#### 2.12.2 Spread plate method

MRSA (Gram-positive) and *E. coli*, *K. pneumoniae* and *P. aeruginosa* (Gram-negative) bacteria were used for antibacterial activity evaluation using colony counting on nutrient agar plates (NA).^27^ An equal mixture of Ti-Cu suspension (final concentration 30, 100, 300, 1000 and 3000 µg mL^−1^) with bacterial suspension in MHB (5 × 10^5^ CFU mL^−1^) were incubated in shaker incubator at 37 °C for 3 hours. Then the mixture was irradiated with 1.5 W cm^−2^ NIR light 808 nm for 10 min followed by cultivating 20 µL aliquot of 100 times diluted suspension on NA agar plate at 37 °C for 16–18 h. During laser irradiation, the temperature increase was recorded by thermal imaging system. Afterward, the number of colony forming units and bactericidal rate was evaluated.

### 2.13. Animal study

Female BALB/c mice (age: 6–8 weeks and weight: 14–20 g) with breast cancer were housed at the Animal Center of Zanjan University of Medical Sciences (Iran). The study protocol was approved by Zanjan University of Medical Sciences, Iran (IR.zums.AEC. 1402.034).

The animals were randomly divided into four experimental groups (*n* = 5 per group) consisting of a control group receiving no treatment, a Ti-Cu NCs group administered twice *via* intratumoral injectionof Ti-Cu NCs at a dose of 250 µg mL^−1^, an NIR group exposed to NIR without Ti-Cu NCs, and a combination group receiving both Ti-Cu NCs and NIR. The progression of tumor size, body weight, and physiological behaviors of the animals were regularly monitored throughout the treatment period.

#### 2.13.1 *In vivo* toxicity evaluation

To assess the biosafety of Ti-Cu, healthy female BALB/c mice (6–8 weeks old, weighing approximately 14–20 g) were randomly assigned into two groups: a control group and a Ti-Cu-treated group, with one mouse per group (*n* = 1). The Ti-Cu-treated mouse received a single intraperitoneal injection of 200 µL of Ti-Cu suspension at a concentration of 2 mg mL^−1^. The control mouse received an equivalent volume of sterile PBS *via* the same route. Following injection, both mice were monitored daily for clinical signs of distress, behavioral changes, or weight loss. At 14 days post-injection, the kidneys, liver, spleen, heart, lungs, and brainwere carefully excised. Tissues were fixed in 4% paraformaldehyde, embedded in paraffin, and stained with hematoxylin and eosin (H&E) for histological examination.

#### 2.13.2 Hemolysis assay

The hemocompatibility of Ti-Cu was evaluated using a hemolysis assay.^28^ Fresh blood was collected from healthy mice into tubes containing an anticoagulant. Red blood cells (RBCs) were isolated by centrifugation at 1500 rpm for 10 minutes, washed three times with sterile PBS, and diluted to a 5% (v/v) RBC suspension. The RBC suspension was incubated with various concentrations of Ti-Cu (62.5–1000 µg mL^−1^) at 37 °C for 15, 30, 60, 120, and 240 minutes, as well as 24 hours. PBS and deionized water were used as negative and positive controls, respectively. After incubation, samples were centrifuged at 3000 rpm for 5 minutes, and the supernatant absorbance was measured at 540 nm using a microplate reader. The hemolysis percentage was calculated using the following formula:Hemolysis (%) = [(sample absorbance − negative control absorbance)/(positive control absorbance − negative control absorbance)] × 100.

### 2.14. Histopathological analysis

On days 7 and 14, the lung, spleen, heart, liver, kidney and tumor tissues were collected and fixed with 10% formalin for H&E staining.The organs were dehydrated, clarified with xylene and paraffin-embedded, sectioned using a microtome, and stained using the H&E method. Histopathological examination was performed using a microscope, Dino-Lite camera, and Dino-Capture software (V.2).^29^

### 2.15. Statistical analysis

Data analyses were performed with GraphPad Prism 8.0. The experimental results were presented as mean ± SD (standard deviation). A *t*-test and ANOVA were used to calculate significant difference between different groups. *p < 0.05, **p < 0.01, ***p < 0.001, and ****p < 0.0001 were considered statistically significant.

## 3. Results and discussion

### 3.1. Comprehensive characterization analysis of Ti-Cu: morphological and chemical insights

**Fig. 1:**
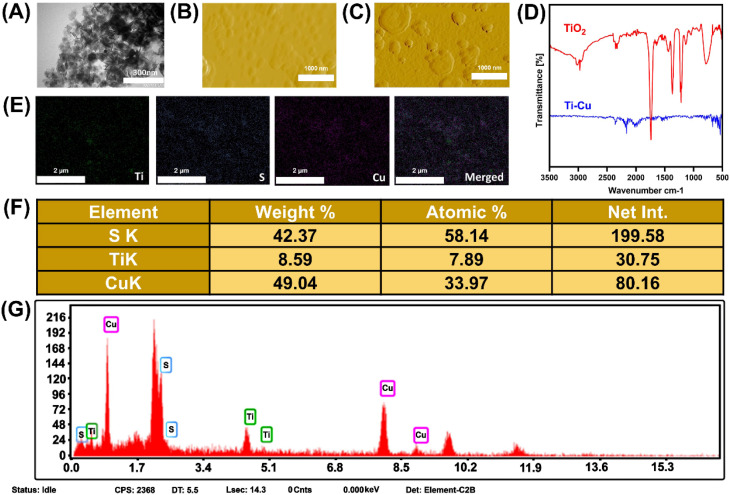
Characterization of the synthesized Ti-Cu nanocomposite: (A) TEM image showing aggregated nanoscale particles (scale bar: 300 nm). (B and C) AFM images illustrating the surface morphology and topography of the nanocomposite (scan area: 2 µm × 2 µm). (D) ATR FTIR spectra of TiO_2_ and Ti-Cu nanocomposite. (E) EDX quantitative elemental analysis. (F) Elemental composition spectrum. (G) Elemental mapping images showing the spatial distribution of Ti, Cu, and O.

TEM analysis (Fig. 1A) revealed the formation of nanoscale particles with an irregular morphology, exhibiting a tendency to form aggregated clusters. The average size of the nanocomposite based on TEM results was 64.02 ± 10.03 nm. (Fig. S6) This observation aligns with previous reports on Cu-doped TiO_2_ nanoparticles, suggesting the presence of interconnected domains characteristic of metal oxide nanostructures formed to minimize high surface energy and strong interparticle interactions. Such aggregation behavior is commonly attributed to the reduction of surface energy during nanoparticle synthesis and subsequent processing stages.^30,31^ The surface morphology was further examined using AFM (Fig. 1B and C). The AFM micrographs present a rough and heterogeneous surface topography, characterized by aggregated nanoscale grains forming irregular clusters. This nanostructured framework indicates significant surface roughness, a common feature in nanomaterials resulting from interparticle forces and high surface area requirements.^32^ Elemental composition and distribution were investigated using EDX spectroscopy. The EDX spectrum (Fig. 1F) confirmed the principal elements present in the synthesized material as titanium (Ti), copper (Cu), and oxygen (O). Quantitative analysis presented in the EDX data (Fig. 1E) supports the successful formation of the Ti-Cu nanocomposite, with no significant impurity elements detected. Elemental mapping images (Fig. 1G) illustrate the spatial distribution of Ti, Cu, and O across the analyzed area, indicating a relatively homogeneous dispersion of Cu species within the Ti-based matrix.^33^

Chemical bonding and surface functional groups were analyzed by ATR-FTIR spectroscopy (Fig. 1D). The spectrum, recorded in the range of 400–4000 cm^−1^, shows a weak absorption band around 2970 cm^−1^ attributed to C–H stretching vibrations, indicative of minor residual organic species from the synthesis process. A distinct band at 1738 cm^−1^ is assigned to C=O stretching vibrations. Additional bands at 1366 and 1216 cm^−1^ are attributed to C–O stretching vibrations or Ti–O–C related bonds. A band near 786 cm^−1^ may correspond to Ti–O–Ti lattice vibrations. The most prominent absorption features, observed between 670 and 420 cm^−1^, are characteristic of Ti–O stretching and Ti–O–Ti bridging vibrations within the titanium oxide lattice, consistent with findings for TiO_2_-based nanostructures.^34,35^

#### 3.1.1 Evaluation of photothermal and photocatalytic properties of Ti-Cu NC

The photothermal and photocatalytic performance of the Ti-Cu nanostructures was systematically evaluated. The incorporation of copper into the TiO_2_ lattice significantly alters the electronic structure, leading to enhanced visible-light harvesting and improved charge carrier dynamics, which are critical for both heat conversion and catalytic efficiency.^36^

**Fig. 2:**
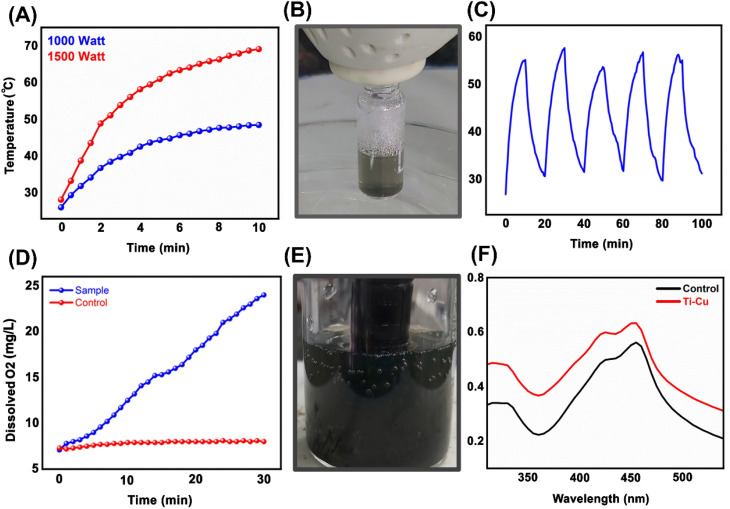
Photothermal performance evaluation of the Ti-Cu. (A) Temperature curves of the Ti-Cu sample under irradiation at different power densities (1 and 1.5 W cm^−2^). (B) Gas production bubbles for 1 mg mL^−1^ Ti-Cu. (C) Photothermal stability test showing temperature evolution over five on/off irradiation cycles. (D) Dissolved oxygen (O_2_) concentration measurements for the Ti-Cu sample and the control, (E) nanoparticle dispersion in aquas solution (F) UV-vis absorption spectrum.

As shown in Fig. 2A, the Ti-Cu sample exhibited a robust photothermal response. Under 1.5 W cm^−2^ irradiation, the temperature rose from 28 °C to approximately 68 °C within 10 minutes, demonstrating high light-to-heat conversion efficiency. The photothermal conversion efficiency of Ti-Cu was measured to be 34.26%. It should be noted that we have calculated this value in many of our previous publications.^4,29,37^ While pure TiO_2_ is widely recognized as a photocatalyst in photodynamic therapy rather than an efficient photothermal material due to its wide band gap (∼3.0–3.2 eV), where absorbed photon energy is mainly utilized for the generation of electron–hole pairs instead of heat production. This value has been reported about 30.9% in a similar nanocomposite which is comparable with our Ti-Cu composite.^38^ In addition, there is a study reporting carbon-decorated copper nanoparticles, in which the photothermal conversion efficiency was reported to be 48.5%,^39^ which is higher than the value reported in our study. This difference can be attributed to the presence of carbon, as carbon itself can also exhibit photothermal activity. In a study in which only copper nanoparticles were used, this value was lower (23.9%^40^) than the value obtained in our work. This difference can be attributed to the presence of titanium in our composite. Moreover, in one of our previous studies, in which copper–cysteine was used, this value was reported to be 22.02%.^41^

This rapid thermal elevation is attributed to the non-radiative recombination of photogenerated carriers and localized surface plasmon resonance (LSPR) effects induced by Cu species.^42^ Furthermore, the stability of this conversion was confirmed through five successive on/off cycles (Fig. 2C), where the maximum temperature remained consistent between 55–60 °C, proving the material's structural integrity and thermal durability.

The photocatalytic oxygen evolution reaction (OER) was monitored *via* dissolved oxygen (DO) levels (Fig. 2D). The Ti-Cu nanostructures achieved a DO concentration of approximately 23 mg L^−1^ after 30 minutes of illumination, significantly outperforming the control. This production of oxygen is visually supported by the formation of gas bubbles in the reaction vessel (Fig. 2B). Such enhancement in OER is typically driven by the lowered bandgap and the trap states created by Cu dopants, which facilitate the oxidation of water molecules.^43^

The UV-vis absorption spectra (Fig. 2F) further elucidate these findings, showing a strong absorption peak at approximately 650 nm for the Ti-Cu sample. This red-shift from the ultraviolet to the visible region confirms the successful modification of the TiO_2_ optical properties through copper doping, directly correlating with the observed increase in photocatalytic and photothermal activity.^36^

### 3.2. Cellular uptake of Ti-Cu is time and dose dependent

**Fig. 3:**
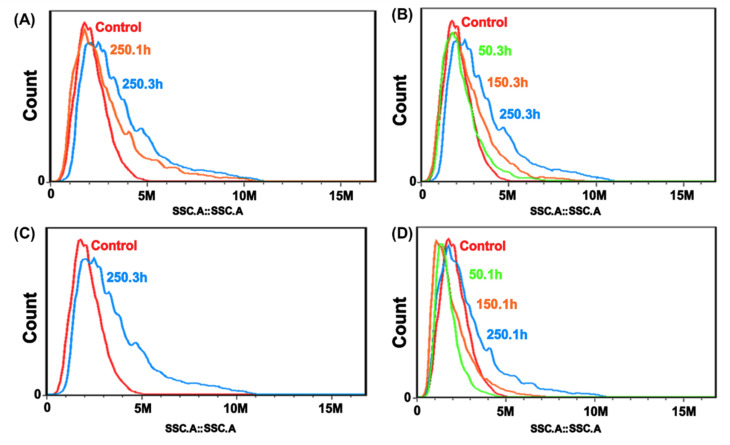
Flow cytometric analysis of Ti–Cu uptake by 4T1 cells. Rightward shifts in SSC-A histograms indicate increased granularity due to nanoparticle internalization. Cells were treated with 50, 150, or 250 µg mL^−1^ Ti–Cu for 1 or 3 h. (A) Untreated control, 250 µg mL^−1^ (1 h), and 250 µg mL^−1^ (3 h). (B) Untreated control *vs* all concentrations (3 h). (C) Untreated control *vs* 250 µg mL^−1^ (3 h). (D) Untreated control *vs* all concentrations (1 h). Data are representative of three independent experiments.

Ti-Cu uptake by 4T1 cells was evaluated by flow cytometry analysis using side scatter (SSC) measurements, as increased cellular granularity correlates with particle internalization.^26^Fig. 3 represents histograms related to the side scatter (SSC-A) profiles of 4T1 cells after 1 and 3 hours of exposure to Ti-Cu NCs at 50, 150 and 250 µg mL^−1^. The dose and time evaluation graphs are shown in separate panels for clarity. Treatment with 250 µg mL^−1^ resulted in a mean intensity of 3.53, compared to 2.13 in the control group (Fig. 3C). The rightward shift in the SSC-A peak over time, relative to the untreated control, demonstrates an increase in cellular granularity, indicating progressive particle internalization.

Cells were incubated with increasing concentrations of Ti-Cu particles (50, 150 and 250 µg mL^−1^) for 3 hours. Following this incubation, a significant SSC shift was observed as the dose increased from 50 to 250 µg mL^−1^ (Fig. 3B). In summary, the histograms and quantitative analysis demonstrated that the cellular uptake of the nanocomposite into 4T1 cells was both time (Fig. 3A) and concentration (Fig. 3B and D) dependent.^26^

### 3.3. The Ti-Cu NC caused ROS formation, cell death and apoptosis in the 4T1 cell line, a synergic effect with NIR irradiation

**Fig. 4:**
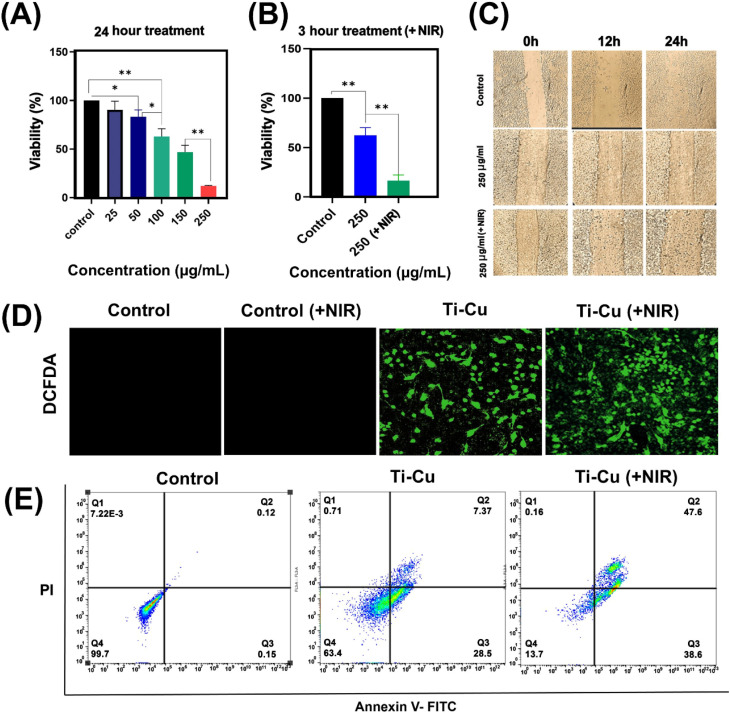
*In vitro* therapeutic evaluation of Ti-Cu against 4T1 breast cancer cells. (A) The percent viability of 4T1 cells treated with different concentrations of Ti-Cu NPs ± NIR (1.5 W, 5 min) after 24 and 3 hours in the MTT assay. Data are presented as mean ± SD (*n* = 3). *P < 0.05, and ***P < 0.001. (C) Migration assay of 4T1 cells treated with 250 µg mL^−1^ Ti-Cu NPs with or without NIR (1.5 W, 5 min). (D) Intracellular reactive oxygen species (ROS) generation detected by DCFH-DA fluorescent probe. (E) Flow cytometric analysis of apoptotic 4T1 cells incubated with 250 µg mL^−1^ Ti-Cu NPs with or without NIR (1.5 W, 5 min) using Annexin V-FITC/PI double staining.

The cytotoxicity of Ti-Cu NCs against 4T1 breast cancer cells was evaluated by MTT assay. While TiO_2_ had no significant toxicity effect on cells (in any dose and exposure time, SI Fig. S1), doping Cu on TiO_2_ nanocomposite caused significant and dose-dependent cytotoxicity (Fig. 4A). Cell viability decreased from 95% at 25 µg mL^−1^ to 10% at 250 µg mL^−1^ after 24 exposures of Ti-Cu. This enhanced cytotoxicity can be attributed to the modulation of physicochemical properties of TiO_2_ nanoparticles through Cu doping. It has been demonstrated that Cu doping induces the oxidative stress-mediated cytotoxicity of TiO_2_ nanoparticles, as evidenced in A549 lung cancer cells. The incorporation of copper not only alters the surface chemistry and electronic structure of TiO_2_ but also promotes ROS generation, leading to irreversible cellular damage and apoptotic cell death.^16,44^

However, no statistically significant difference was observed between cells treated with Ti-Cu with or without NIR irradiation (SI Fig. S2), indicating masking of any potential synergistic effect under prolonged Ti-Cu exposure. To overcome this, concentration and treatment period were selected based on flow cytometric cellular uptake analysis, which revealed that cellular internalization of Ti-Cu was time and concentration dependent, with the highest uptake observed at 250 µg mL^−1^ following 3 hours of incubation (Fig. 3) and mild Ti-Cu toxicity as observed (Fig. 4B). Accordingly, this concentration and time point were selected to evaluate the synergistic effect of Ti-Cu and NIR. Under these optimized conditions, a synergistic enhancement in cytotoxicity was observed upon NIR irradiation, demonstrating the photothermal effect of the Ti-Cu nanocomposite (Fig. 4B).

The intracellular ROS generation was detected following Ti-Cu treatment with or without NIR, detected by DCFH-DA staining and as seen in Fig. 4D and SI S3. The intensity of green fluorescence increased in the cells treated with Ti-Cu ± NIR compared to untreated cells showing a significant difference in ROS levels. These results provided further insight into the mechanisms underlying the cytotoxic effects of Ti-Cu.^16^

To further evaluate anticancer efficacy, Ti-Cu with or without NIR treated 4T1 cells were stained with Annexin-FITC/PI to explore apoptotic cells. Ti-Cu treatment induced total early and late apoptotic cells up to 35%. NIR irradiation has increased this rate to 86%. Combination of Ti-Cu and NIR irradiation significantly elevated both early and late apoptotic populations (Fig. 4E). These findings demonstrate that the synergistic effect of Ti-Cu with NIR effectively promotes apoptotic cell death in 4T1 cancer cells. The cytotoxicity induced by Ti-Cu is mechanistically linked to the generation of ROS and the subsequent activation of apoptotic pathways.^25,45,46^ Copper-doped titanium dioxide nanoparticles have been shown to induce oxidative stress in a dose-dependent manner, as evidenced by increased ROS production, depletion of glutathione (GSH), and a reduction in mitochondrial membrane potential (MMP) in human lung epithelial (A549) cells.^44^ The photothermal effect induced by NIR irradiation provides an additional and synergistic mechanism for enhancing cancer cell death. Upon NIR irradiation, photothermal agents convert absorbed light energy into localized hyperthermia, generating heat that raises the temperature within the tumor microenvironment.^47^ This localized heating not only directly ablates cancer cells but also amplifies the cytotoxic response through multiple pathways. The heat generated during PTT has been shown to intensify oxidative stress by further increasing ROS production, leading to more severe mitochondrial dysfunction and membrane damage.^48^

### 3.4. Ti-Cu NCs reduced the migratory capacity Of 4T1 cells

A scratch assay was done to assess the impact of Ti-Cu with or without NIR on the cellular migration of 4T1 cells. Results showed a significant reduction in gap filling compared to the control and non-treated group. These results demonstrate the inhibitory effect of Ti-Cu on 4T1 cell migration. It is important to note that due to the Ti-Cu toxicity observed in prolonged exposure, no observable difference was found between the Ti-Cu group and the Ti-Cu with NIR illumination group. While the control cells proliferated significantly and filled the scratch area over 24 hours, the Ti-Cu-treated cells (with or without NIR) lost this ability and, with increased treatment duration, detached from the cell culture plate surface.

### 3.5. Ti-Cu with NIR irradiation induces key indicators of immunogenic cell death in 4T1 cells

**Fig. 5:**
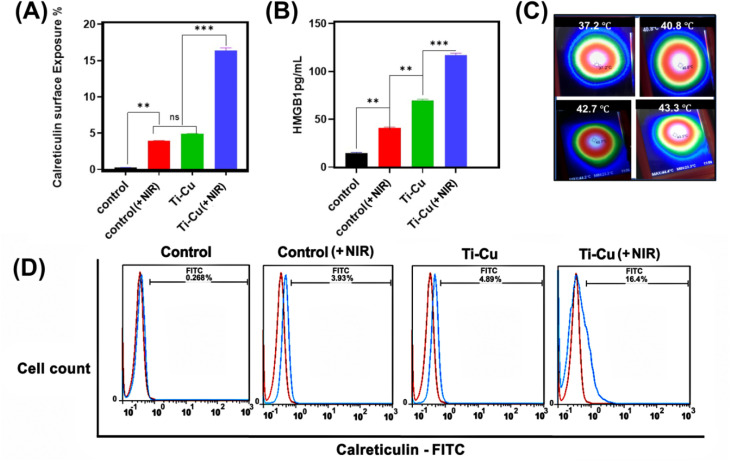
Immunogenic Cell Death (ICD) induction in 4T1 breast cancer cells following Ti-Cu NC treatment with and without NIR irradiation. (A) Quantitative analysis of calreticulin (CRT) surface expression on 4T1 cells. (B) Extracellular release of HMGB1 (high mobility group box 1) in 4T1 cell supernatants. (C) Photothermal performance of Ti-Cu on 4T1 cells treated with Ti-Cu upon NIR irradiation for 5 min. (D) Flow cytometric analysis of cell surface calreticulin (CRT) exposure in 4T1 cells treated with Ti-Cu ± (NIR). Representative histograms show CRT staining (blue curves) overlaid with their respective unstained or isotype controls (red curves).Data are representative of three independent experiments. *P < 0.05, and ***P < 0.001.

The induction of ICD in 4T1 cancer cells following Ti-Cu NCs treatment was evaluated through detection of key damage -associated molecular patterns (DAMPs).^48^ As shown in Fig. 5A, the results revealed that Ti-Cu with NIR irradiation significantly increased calreticulin surface expression compared to the control, while Ti-Cu treatment induced moderate elevation. Representative flow cytometry histograms (Fig. 5D) show this, with CRT-positive cells increasing from 0.268% in the control group to 16.4% in Ti-Cu with NIR treated cells. This pronounced increase in surface exposure of CRT suggests that Ti-Cu-mediated photothermal activation by NIR effectively promotes ICD associated signaling in 4T1 cells.^49^

Furthermore, another key marker of ICD, extracellular high-mobility group box 1 (HMGB1) was quantified in the culture supernatants of treated 4T1 cells by ELISA (Fig. 5B). Untreated control cells released a baseline level of HMGB1 (14 pg mL^−1^), while NIR irradiation induced a modest increase (41 pg mL^−1^). Treatment with Ti-Cu in the absence of irradiation resulted in a more pronounced release (69 pg mL^−1^). HMGB1 was significantly elevated in the Ti-Cu with NIR group. Notably, the combination of Ti-Cu with NIR irradiation triggered the most substantial HMGB1 release (117 pg mL^−1^). This synergistic enhancement, which aligns with the observed CRT surface exposure, indicates that NIR-activated Ti-Cu NSs robustly induce ICD markers in breast cancer cells.^49–51^

Recent studies have demonstrated that copper-based nanomaterials can trigger ICD through multiple pathways, including generation of ROS *via* Fenton-like reactions, endoplasmic reticulum stress and mitochondrial dysfunction^52^ leading to CRT exposure and HMGB1 release.^53^ The significant enhancement of ICD markers following NIR irradiation can be attributed to the photothermal properties of Ti-Cu component, which generates localized hyperthermia as shown in Fig. 5C (43.3 °C) sufficient to induce heat shock protein expression and facilitate DAMP release.^53^ The release of HMGB1 is particularly significant, as this nuclear protein, translocate from nucleus to the cytoplasm for extracellular release,^54^ acts as a Toll-like receptor 4 (TLR4) agonist, promoting dendritic cell maturation and subsequent T-cell activation,^54,55^ ultimately transforming the immunosuppressive tumor microenvironment into an immunogenically active state.

### 3.6. Antibacterial activity

**Fig. 6:**
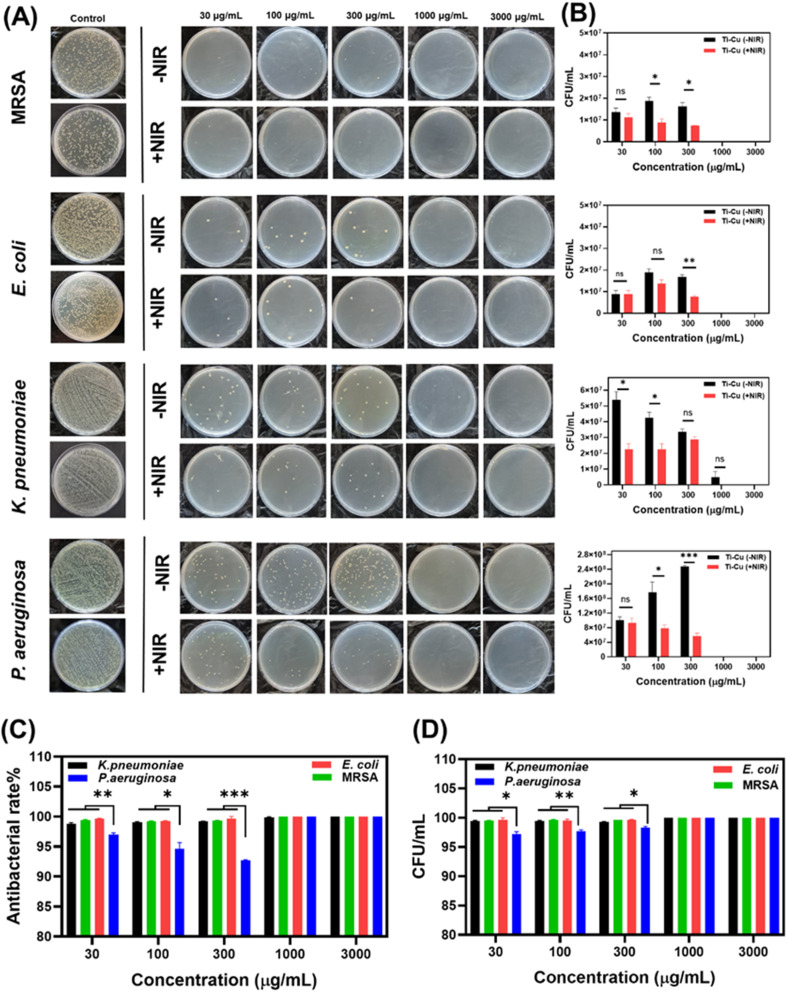
The antibacterial activity of Ti-Cu NC. The antibacterial activity of Ti-Cu against MRSA (Gram-positive) and *E. coli*, *K. pneumoniae* and *P. aeruginosa* (Gram-negative) with or without NIR. (A) Colony forming unit decrease related to tested Ti-Cu different concentrations (30–3000 µg mL^−1^) (B), and antibacterial rate without (C) and with (D) NIR irradiation is shown.

The antibacterial activity of Ti-Cu NC was evaluated against four most prevalent bacteria in cancer patients including MRSA, *E. coli*, *K. pneumoniae* and *P. aeruginosa*,^56–58^ considering different concentrations of Ti-Cu (30–3000 µg mL^−1^) in contact with these bacteria after 3 hours of incubation. The results are shown in Fig. 6. Normal saline (0.9% W/V NaCl) was used as the control.

For MRSA, the antibacterial activity was more than 99% and at concentrations higher than 1000 µg mL^−1^, the antibacterial activity reached 100%. NIR treatment causes more a more than two-fold decrease in MRSA colony-forming units (CFU) at concentration of 100 and 300 µg mL^−1^. It is assumed that strong electrostatic attraction of positive nanoparticles and surface of MRSA (with negative charges) is responsible of such high mortality. For *E. coli*, 2 fold decrease in colony counting unit was detected at concentration of 300 µg mL^−1^. Significant decrease (*p* < 0.001) in colony counting unit of *P. aeruginosa* (more than four-fold) was observed after NIR irradiation at concentration of 300 µg mL^−1^. Lower antibacterial rate of *P. aeruginosa* compared to the other bacteria (Fig. 6C) is due to its higher resistance. Antibacterial rate of *P. aeruginosa* increases from 97% at 300 µg mL^−1^ to 99.99% at concentration of 1000 µg mL^−1^ of nanoparticle. Increased mortality of *P. aeruginosa* with diverse resistance genes seems a great achievement. There are so limited studies indicating the antibacterial activity of Ti-Cu nanoparticles against *P. aeruginosa*. In addition, NIR treatment showed decrease in count of *K. pneumoniae* at all tested concentrations demonstrating the sensitivity of Gram-negative bacterial outer membrane and lipopolysaccharide to NIR irradiation. Although MRSA and *E. coli* are the most frequently studied bacteria in cancer patients, the antibacterial activity of Ti-Cu nanoparticle against *K. pneumoniae*, the most frequent bacterial infection in bloodstream of cancerous cases, has not been reported previously.^57–59^

Antibacterial rate of four groups of bacteria without and with NIR irradiation is shown in Fig. 6C and D, respectively. The antibacterial rate of 99.57%, 99.4%, 99.6% and 98.3% were recorded for MRSA, *K. pneumoniae*, *E. coli* and *P. aeruginosa* at concentrations of 300 µg mL^−1^, respectively. At concentration of 1000 µg mL^−1^, the antibacterial rate was 100% for all four pathogenic strains in presence of NIR. These results demonstrated the broad spectrum of antibacterial activity of Ti-Cu, and bacterial suppressed suppression through the synergistic activity of the photothermal effect of Ti-Cu at lower concentrations than 1000 µg mL^−1^.

### 3.7. *In vivo* anticancer efficacy of Ti-Cu with NIR irradiation

**Fig. 7:**
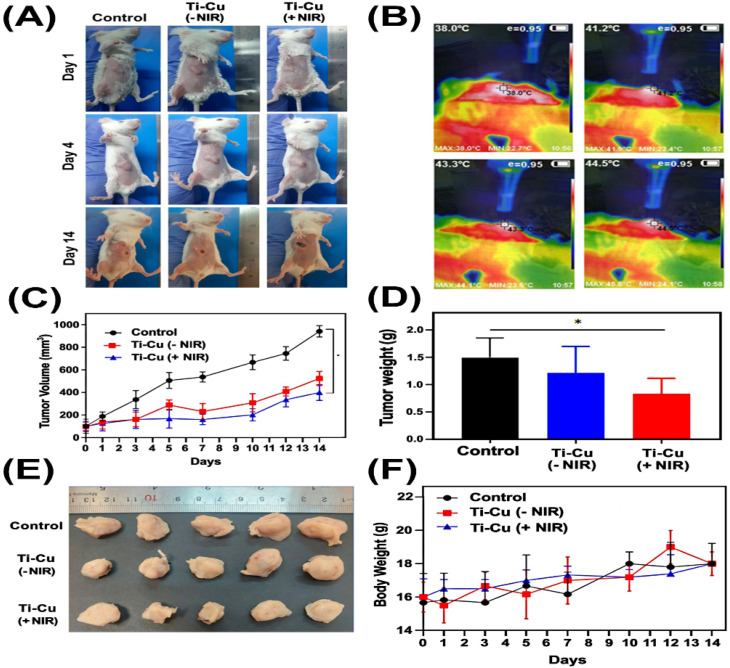
*In vivo* therapeutic efficacy of Ti-Cu in tumor-bearing mice. (A) Representative photographs of each treatment group at days 1, 4, and 14. Mice were treated with either PBS or Ti-Cu ± NIR. (B) Thermal images of tumor-bearing mice following intratumorally injection of Ti-Cu and exposure to NIR laser irradiation, show progressive heating of the tumor region, with maximum temperatures reaching 44.5 °C, in tumor. Control mice show minimal temperature elevation (maximum 2.7 °C) under identical imaging conditions. (C) Final tumor volume (D) tumor weight (E) tumor size in treatment groups. (F) Body weight changes throughout the 14 day treatment period across all experimental groups. Data are presented as mean ± SD (*n* = 5 per group). *p < 0.05, compared to control group.

The efficacy of Ti-Cu NCs in combination with NIR and their therapeutic potential were assessed at *in vivo* setting using a breast tumor model. The mice were subjected to intratumorally injection of 250 µg mL^−1^ NCs and, then irradiated with NIR at 1.5 W cm^−2^ as shown in Fig. 7B, infrared thermal imaging revealed that intratumoral injection of Ti-Cu followed by NIR irradiation induced localized temperature elevation, reaching maximum temperatures of 43.3 °C to 44.5 °C at the tumor site, while control mice exhibited minimal temperature change (maximum 2.7 °C). This efficient photothermal conversion is attributed to the copper doping within the TiO_2_ matrix, which enhances near-infrared absorption thereby generating localized hyperthermia sufficient for tumor ablation.^13^ This targeted heat generation at the tumor site (Fig. 7B) minimizes thermal damage to surrounding healthy tissues, addressing a critical safety consideration for photothermal therapy applications.^60^ As shown Fig. 7C–E, Ti-Cu treatment combined with NIR irradiation resulted in significant reduction in tumors weight and volume compared to the control group.

The moderate tumor suppression observed in Ti-Cu treatment, aligns with recent reports demonstrating that Cu doped TiO_2_ nanocomposites exhibit intrinsic cytotoxicity through ROS generation and copper ion release, achieving approximately 47–72% cancer cell death *in vitro* even without photoactivation.^44^

However, the significant enhancement following NIR irradiation confirms the synergistic effect of photothermal therapy combined with Ti-Cu nanoparticles. Importantly, body weight monitoring (Fig. 7F) revealed no significant weight loss across any treatment group throughout the experimental period, indicating minimal systemic toxicity and favorable biosafety profile as confirmed by the *in vivo* toxicity assessment and hemolysis assay (Fig. 9).

#### 3.7.1 Histopathological analysis

**Fig. 8:**
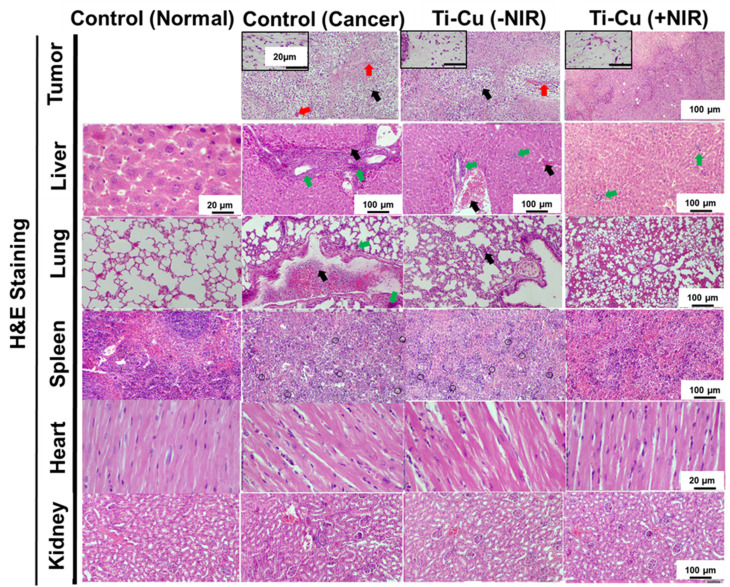
Histopathological analysis of major organs from tumor-bearing Mice following Ti-Cu ± NIR. Representative H&E staining of staining images of excised tumor, the kidney, the liver, the lung, the spleen, and the heart from different groups at day 14. Angiogenesis, inflammatory cells, metastatic nodules, are shown as red, black, and green arrows, respectively. The black hollow circles show the polps in the Spleen.

The H&E staining was used to study tumor treatment by the Ti-Cu nanocomposite and metastasis to the lung and the liver (Fig. 8). Consistent with the tumor growth data, the control and Ti-Cu (−NIR) groups showed abundant tumor and inflammatory cells was observed in the tumor area as well as increased angiogenesis. In accordance with tumor progressing, the H&E staining showed that the Ti-Cu (+NIR) induced the obvious tumor cell necrosis with decreased angiogenesis, further verifying that the Ti-Cu nanocomposite could destroy the tumor cells through the photothermal effect. The above excellent results clearly confirmed that nanocomposite have great potential for *in vivo* photothermal ablation of tumors.

The excellent *in vivo* antitumor activity of Ti-Cu (+NIR) encouraged us to further evaluate its potency as anticancer metastasis agent. As shown in Fig. 8, liver metastases were clearly observed in the control group Ti-Cu (−NIR) groups. In addition, plenty of inflammatory cells were observed in the hepatic vessels (black arrows). In contrast, metastatic nodules were remarkably decreased in Ti-Cu (+NIR) group (green arrows). The Lung of control group had many pulmonary metastatic nodules. In addition, numerous inflammatory cells were observed within pulmonary blood vessels. In the case of Ti-Cu (−NIR) lung tissue had low inflammatory cells and metastatic nodules. No pulmonary metastatic nodules and a few inflammatory cells were observed in Ti-Cu (+NIR) group than the other groups. As shown in the H&E staining such pulp was considerably decreased. No abnormality was seen in the heart the kidney tissues of the treated groups.

#### 3.7.2 *In vivo* biosafety evaluation

**Fig. 9:**
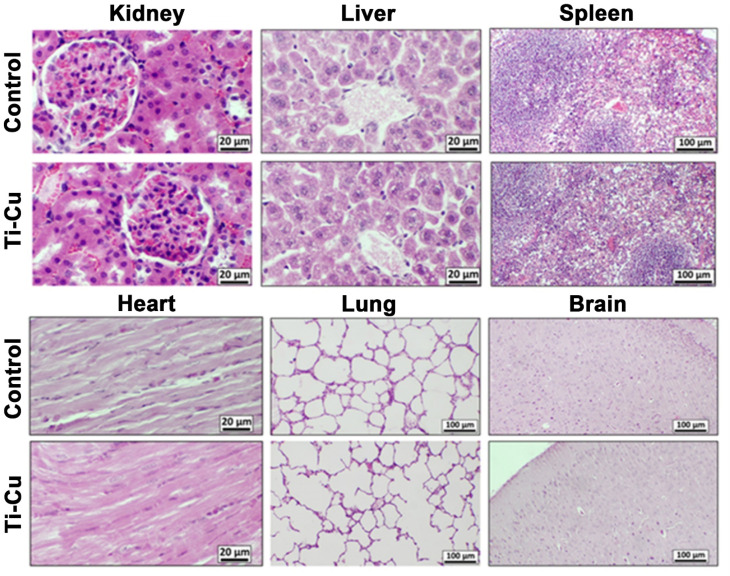
*In vivo* toxicity evaluation of Ti-Cu in mice. Representative histological images (H&E staining) of major organs including kidney, liver, spleen, heart, lung, and brain—harvested from mice 14 days post-intraperitoneal injection of either PBS (control) or 2 mg of Ti-Cu nanoparticles. Sections were imaged at 20× magnification with scale bars representing 20 µm or 100 µm as indicated.

*In vivo* biocompatibility is of vital importance for the NPs applied in drug delivery systems.^61^ Biocompatible properties of Ti-Cu were evaluated by IP injection of the NCs in normal Balb/c mice (Fig. 9). The main organs including Brain, Heart, Kidney, Liver, Lung, and Spleen were harvested and assessed by H&E staining on day 14. As shown in H&E images, no obvious pathological abnormalities or inflammation was observed in main organs. In addition, our results showed that the remaining animals had 100% survival after 2 weeks. These findings suggested the that the NCs did not cause toxicity after IP injection with even higher dose of the NCs (200 µL and 2 mg kg^−1^), thus confirming biocompatibility of the NCs.

## 4. Conclusion

In summary, this study demonstrates that Ti-Cu NCs serve as a novel photothermal platform for cancer therapy. Upon NIR irradiation, Ti-Cu NCs induced ROS-mediated apoptosis, suppressed 4T1 cell migration, and triggered key ICD markers (calreticulin exposure and HMGB1 release), indicating potential to activate antitumor immunity against residual and metastatic disease. Concurrently, the nanocomposites exhibited potent synergistic photothermal antibacterial activity against clinically relevant pathogens in cancer patients. *In vivo*, Ti-Cu NCs combined with NIR irradiation showed anticancer efficacy without detectable toxicity, even at elevated doses. These findings introduce Ti-Cu NCs as a promising candidate against cancer recurrence, metastasis, and bacterial complications.

## Conflicts of interest

The authors declare no conflict of interest. They have no known competing financial interests or personal relationships that could have appeared to influence the work reported in this paper.

## Supplementary Material

RA-OLF-D6RA04493J-s001

## Data Availability

The data that support the findings of this study are available from the corresponding author upon reasonable request. Supplementary information (SI) is available. See DOI: https://doi.org/10.1039/d6ra04493j.
