# Supplementary material for: An effective photothermally active titanium-copper nanocomposite for breast cancer therapy
Source: RSC Adv. 2026 Jul 7. Online ahead of print. doi: 10.1039/d6ra04493j (PMC13339989; doi:10.1039/d6ra04493j)
Supplement: RA-OLF-D6RA04493J-s001 [file RA-OLF-D6RA04493J-s001.pdf]

Supplementary Information (SI) for RSC Advances.
This journal is © The Royal Society of Chemistry 2026

Supplementary Information

An Effective Photothermally Active Titanium-Copper Nanocomposite for Breast Cancer Therapy

Laleh Salarilak[1], Abolfazl Doosti[2], Mehdi Haddad[3], Ali Kalantari-Hesari[4] ,Faezeh Almasi[1], Farnaz Eslami[1], kiana Nazari [3], Mir-Jamal Hosseini [5], Aziz Maleki[1,2*]

Affiliations:

[1]Zanjan Pharmaceutical Nanotechnology Research Center (ZPNRC), Zanjan University of Medical Sciences, Zanjan 45139-56184, Iran.

[2]Department of Pharmaceutical Nanotechnology, School of Pharmacy, Zanjan University of Medical Sciences, Zanjan 45139-56184, Iran.

[3]Student Research Committee, School of Pharmacy, Zanjan University of Medical Sciences, Zanjan 45139-56184, Iran.

[4]Department of Basic Sciences, Faculty of Veterinary Medicine, Bu-Ali Sina University, Hamedan, Iran.

[5]Zanjan Applied Pharmacology Research Center, Health and Metabolic Diseases Research Institute, Zanjan University of Medical Sciences, Zanjan, Iran. jamal_hossini@yahoo.com.

*** Department of Pharmaceutical Nanotechnology, School of Pharmacy, Zanjan University of Medical Sciences, Zanjan 45139-56184, Iran. maleki@zums.ac.ir.**

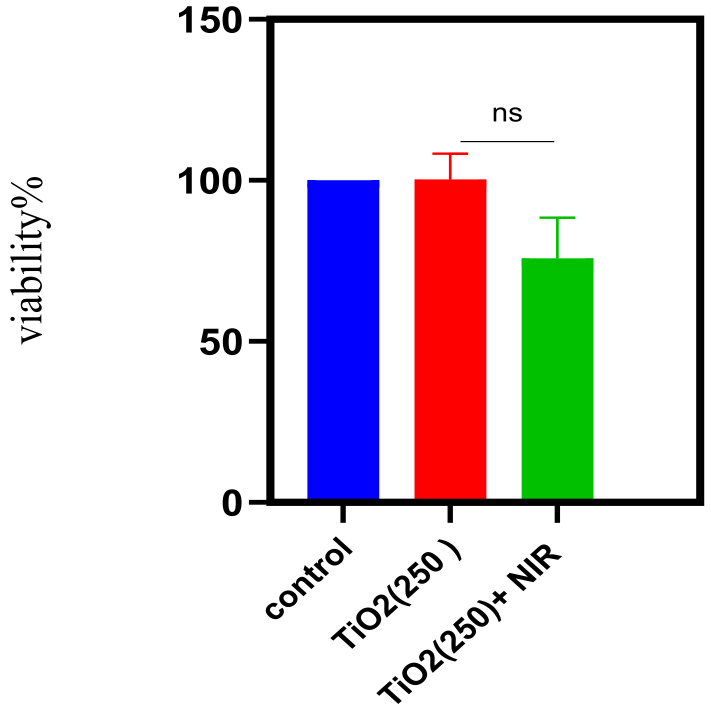

Figure S1. The percent viability of 4T1 cells treated with of Tio_2 ± NIR (1.5 W/cm^2, 5min) after 24.

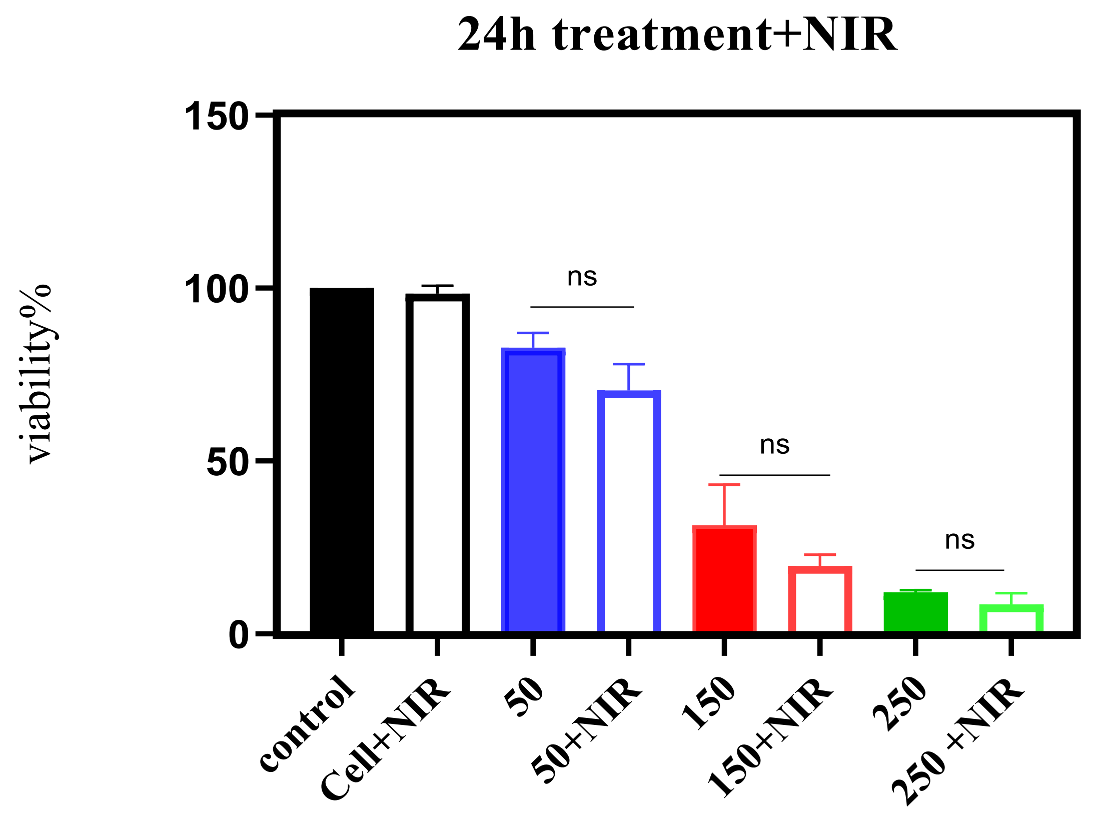

Figure S2. The percent viability of 4T1 cells treated with of Ti-Cu ± NIR (1.5 W/cm^2, 5min) after 24.

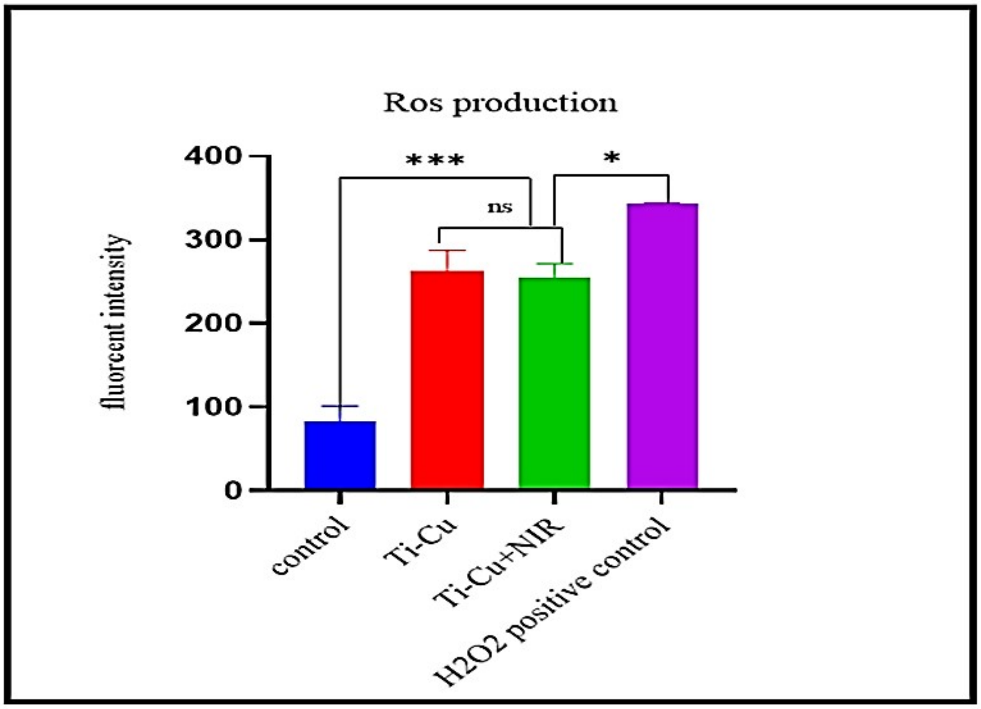

Figure S3. ROS production in 4T1 cells treated with of Ti-Cu ± NIR

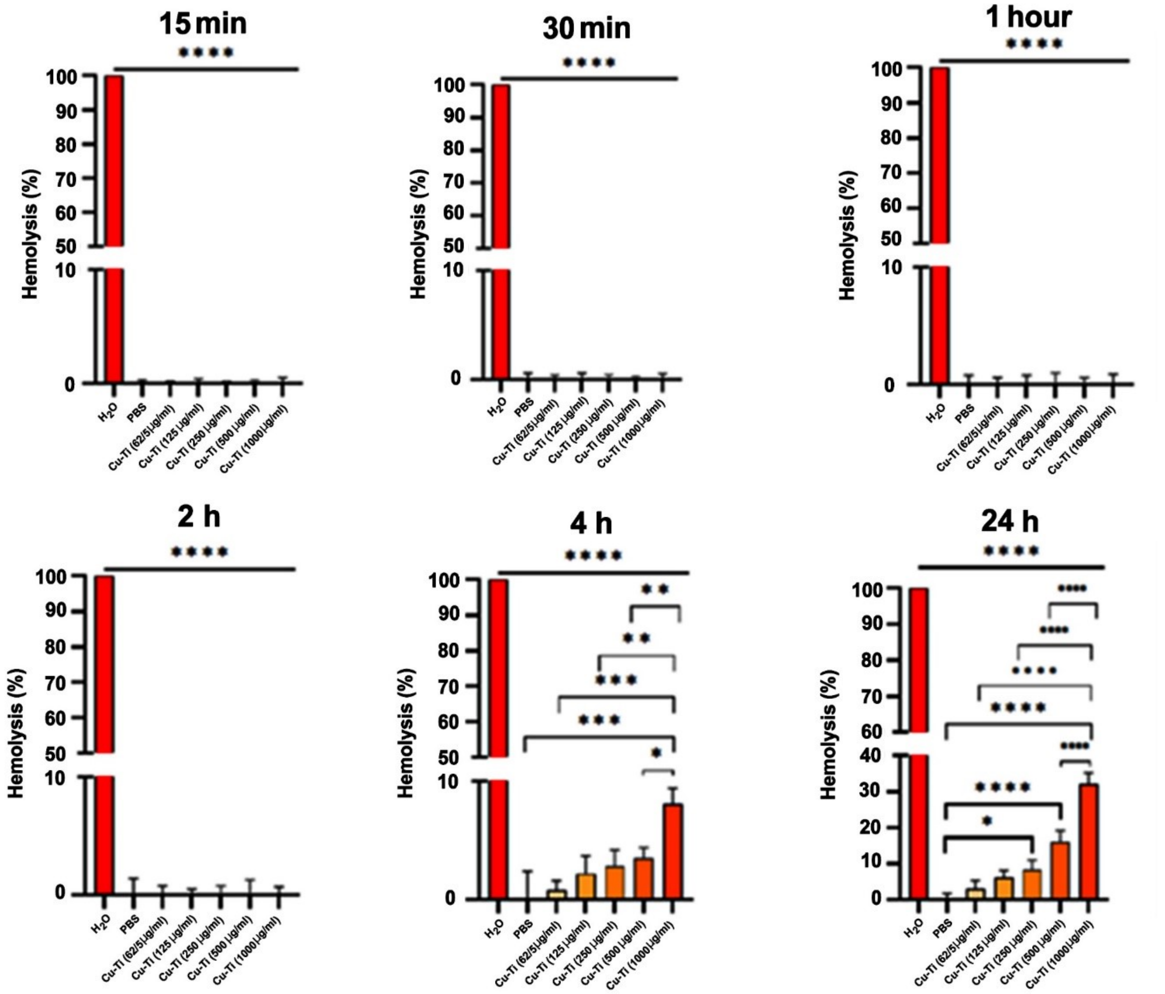

Figure S4. *In vitro* hemocompatibility assessment of Ti-Cu nanoparticles. Red blood cells were isolated and incubated with Ti-Cu nanoparticles at various concentrations and time points (15 minutes, 30 minutes, 1 hour, 2 hours, 4 hours, and 24 hours).

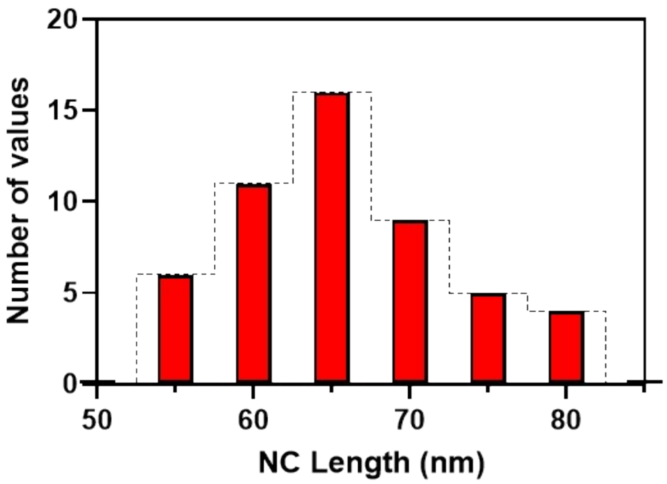

Figure S5. Particle size distribution histogram of the Ti-Cu NC based on TEM image analysis. The average particle size was calculated using ImageJ, yielding a mean diameter of 64.02 ± 10.03 nm (mean ± SD, n = 100).
